# Prosthetic and surgical management of a sizeable epulis fissuratum: a case report

**DOI:** 10.11604/pamj.2022.41.49.31339

**Published:** 2022-01-18

**Authors:** Abdoulmajid Habibou Ibrahim, Nadia Merzouk, Anissa Abdelkoui

**Affiliations:** 1 Department of Removable Prosthodontics, Mohammed V University of Rabat, Faculty of Dentistry, Rabat, Morocco

**Keywords:** Epulis fissuratum, removable prosthesis, tissue conditioning, case report

## Abstract

This case report describes the management of a large-sized Epulis Fissuratum (EF) in a 70-year-old female patient using an original prosthetic technique. It consists of a combination of a tissue conditioning and a resection surgery. The patient´s main concern was a mucogingival tissue growth located on the left mandibular area. The interesting clinical findings deriving from this technique are: tissue conditioning is an important phase prior to the surgical resection of a large EF and occlusal functions can be maintained during the procedures with patient´s corrected dentures. After clinical, radiological and pathological examinations, the diagnosis was of an EF induced by an ill fitted mandibular denture. The intervention carried out was a combination of a 2 weeks tissue conditioning period followed by a cold blade surgical resection. After a 3 months follow-up period, a completely healed vestibular sulcus was observed with no scar tissue. The present technique facilitates the resection surgery by removing the inflammatory component of the EF. It also guides the post-surgical tissue healing, allowing the obtention of a deep vestibular sulcus, thus creating a better bearing surface for subsequent renewed dentures. Not depriving the patient of her dentures during the healing process helped to improve her quality of life and her cooperation.

## Introduction

Epulis fissuratum (EF) is an inflammatory fibrous hyperplasia of the oral mucosa, often located over the alveolar ridges or the soft tissues of the vestibular sulcus [1, 2]. It is a pathological entity that occurs mostly in middle-aged and older subjects with women predilection [3]. Its etiology is multifactorial, associated with poor oral hygiene, smoking, vitamin deficiency, and ill-fitting dentures [4-6]. Clinically, it takes a form of a raised sessile lesion or multiple folds of hyperplasic tissue related to overextended borders/or sharp prosthetic flanges. The lesion is typically firm and fibrous, sometimes erythematous or ulcerated. The EF can be small or in some cases extended to the entire buccal vestibule. It is generally asymptomatic, which explains why the patient continues to wear his/her prosthesis until the lesion becomes important, thus compromising its retention and stability. Certain clinical forms of EF can also be ulcerated. The transformation of such lesion into an oral carcinoma although rare, exists. The diagnosis of the EF is obvious, but an anatomo-pathological confirmation is necessary [7]. The treatment of this lesion, consists on the elimination of the causal factors and the removal of the lesion, either by surgery or by tissue conditioning [1]. This clinical case describes an original technique that includes a combination of a tissue conditioning phase using the corrected patient´s old dentures, then followed by the surgical resection of the EF. It is also essential to note that the post-surgical healing was guided by the corrected dentures, therefore avoiding an eventual recurrence of the lesion.

## Patient and observation

**Patient information:** a 70-year-old female patient was referred to the Department of Removable Prosthodontics of Rabat, Morocco for surgical and prosthetic management of an oral tissue hyperplasia. The patient´s medical history involved: heart disease under anticoagulants; high blood pressure treated with beta blockers and depression. During the first visit, the patient reported that she had been wearing her prostheses for 10 years without any professional prosthetic control, and that a mandibular tissue lesion appeared 1 year ago.

**Figure 1:**
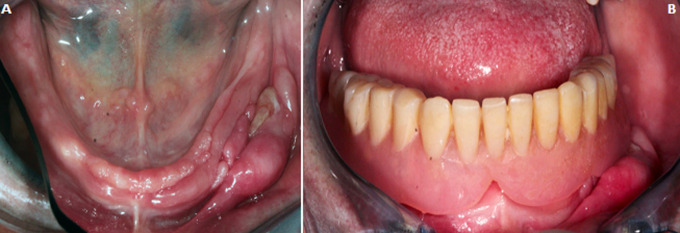
(A) clinical view of mandible and a large epulis fissuratum; (B) ill-fitting mandibular complete denture with an overextended prosthetic border in contact with the hyperplasia

**Clinical findings:** an exo-buccal examination showed a slight diminution of the Vertical Dimension of Occlusion (VDO), and a mandibular pseudo-prognathism. The endo-buccal examination revealed poor oral hygiene, a partially edentulous maxillary arch supporting a provisional partial removable prosthesis and a completely edentulous lower arch supporting a complete denture. At the mandible, a pink to reddish prominent hyperplasia (6.5 cm x 2 cm x 1.7 cm) formed by many sheets in the left mandibular vestibular sulcus and an anterior flabby ridge (Figure 1A). The hyperplasia is non-hemorrhagic during palpation. No cervical lymphadenopathy was evident. Examination of the mandibular denture base revealed a poor maintenance, wear of the artificial teeth and overextended prosthetic borders in contact with the hyperplasia (Figure 1B). On top of that, the mandibular prosthesis does not cover the entire bearing surface. An occlusal examination of the prostheses revealed poorly distributed occlusal contacts.

**Diagnostic assessment:** panoramic radiograph showed a moderate mandibular resorption.

**Diagnosis:** after clinical and radiological examinations, the primary diagnosis was of an epulis fissuratum caused by an ill fitted lower denture and lack of denture maintenance.

**Therapeutic interventions:** a combination of both a tissue conditioning and a surgical excision of hyperplasic tissue. We proceeded as follows:

***Cleaning and correcting the old prostheses:*** correction of the occlusal contacts and prosthetic borders; dentures care and oral hygiene: the patient was advised to brush the oral mucosa and prostheses daily and not to use the dentures at night. A daily massage of the EF for 10 minutes, to accelerate the return of the buccal mucosa to its physiological state.

**Figure 2:**
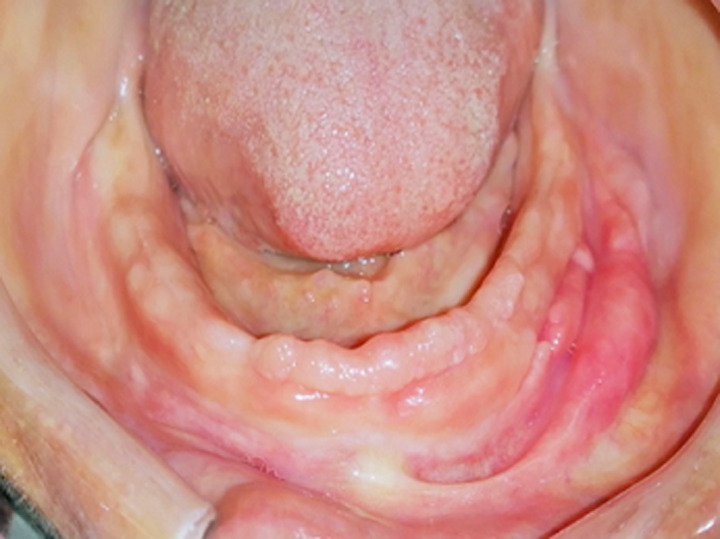
clinical aspect of hyperplasia after three sessions of tissue conditioning

**Figure 3:**
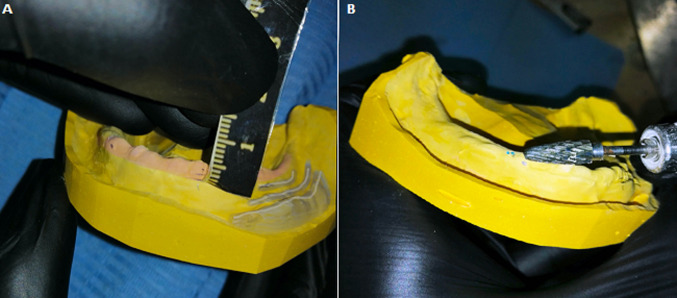
(A) the hyperplasia size measurements reported on the master cast with a pencil; (B) simulation of the surgical excision on the master cast with resin burr

***Tissue conditioning:*** the mandibular denture was loaded with tissue conditioner (GC Soft Liner®) and placed into the patient´s mouth. The patient was then asked to perform all functional movements. The prosthetic borders related to the EF were smoothened by adding and molding the tissue conditioning material so that the prosthetic borders will progressively print a soft and continuous tissue pressure to the EF. The tissue conditioning material was renewed 3 times over the course of 15 days. A significant regression of inflammation and a decrease in size of hyperplasia was noted (Figure 2). At this point a secondary impression under occlusal pressure was taken with a low viscosity polyvinyl-siloxane obtaining a secondary cast on which the measurements of the hyperplasia were reported (Figure 3A).

**Figure 4:**
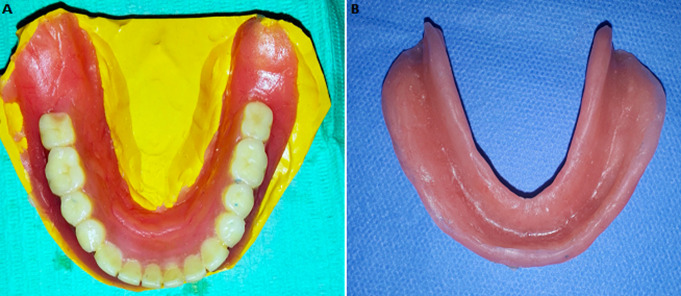
(A) the lower denture was relined with acrylic resin; (B) the mandibular complete denture was rebased with a soft tissue conditioner to allow adequate tissue repair

**Figure 5:**
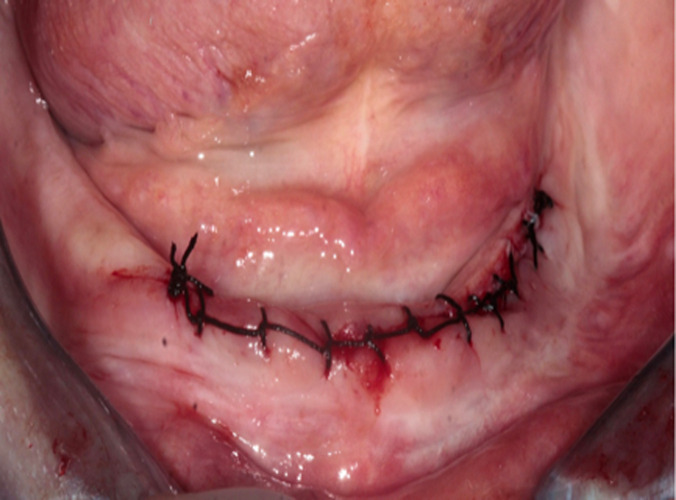
clinical view immediately after the resection surgery

**Figure 6:**
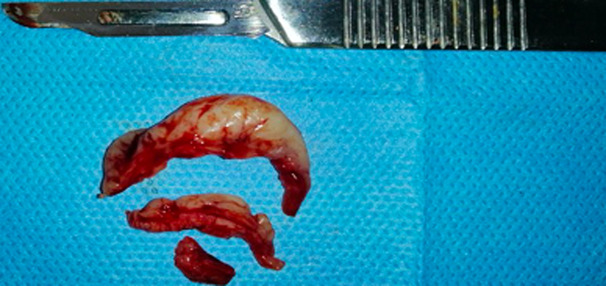
view of the resected tissue, from which a specimen was sampled and sent for anatomopathological examination

In the laboratory, using a burr, a simulation of the surgical excision of the EF and the flabby ridge on the secondary cast was performed (Figure 3B) followed by the relining of the mandibular prosthesis using a self-curing acrylic resin (Figure 4A). The corrected prosthesis was put in place immediately after surgery, to guide the tissue healing and to prevent subsequent surgical revision (Figure 4B). Surgical excision of the flabby ridge and the EF was carried out using a cold blade surgery. (Figure 5). A specimen was sampled after the surgery and sent for anatomo-pathological examination to confirm the primary diagnosis (Figure 6).

**Figure 7:**
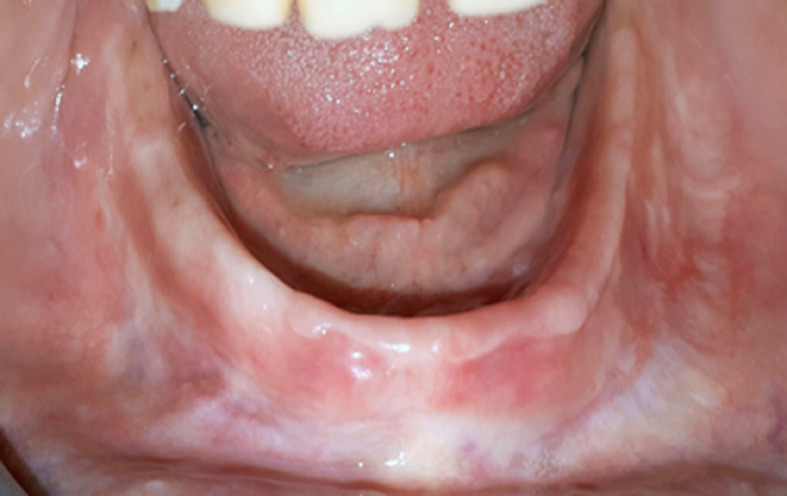
two weeks post-operative view of healing area

**Follow up and outcomes:** the patient was seen after 48 hours to control prosthetic adaptation, and occlusion. After 14 days, substantial tissue healing was observed (Figure 7). After initial bone healing (6 months) new lower and upper prosthesis were fabricated. An annual check-up is prescribed to the patient to detect an eventual recurrence of the EF.

## Discussion

Wearing poorly designed prostheses often leads to tissue damage with different degrees of severity. Epulis fissuratum (EF) is among the most frequently encountered lesions. The treatment of this lesion is necessary before any prosthetic rehabilitation [8].

The treatment of the prosthetic induced EF on a completely edentulous patient, in addition to the elimination of the etiological factors, involves a tissue conditioning and/or pre-prosthetic surgery. The tissue conditioning is a non-invasive therapy which can single-handedly treat clinical cases of small mucosal hyperplasia related to prosthetic wear however, this therapy has certain limitations with extensive lesions with fibrous component which require surgical removal [9].

The most commonly used surgical techniques are: cold blade surgery, electrosurgical surgery, cryosurgery, laser carbon dioxide surgery, Erbium: YAG laser, Neodymium: YAG, or diode laser [10, 11]. In this case, in addition to the elimination of inflammation, the tissue conditioning allowed neuromuscular conditioning of the prosthesis. A surgical excision was performed with a cold blade of the fibrous tissue. A laser surgery could have been used as well as Rezvan. B and colleagues have shown in their study that laser has certain advantages over the conventional surgery such as: absence of tissue contact during surgery, less pain and minimal postoperative edema, shorter healing period and lack of scars [12, 13]. In this protocol, the correction of the existing prosthesis and its use to guide surgery and postoperative healing ensured therapeutic success and prevented recurrence. A comparison of the prosthetic bearing surfaces on the mandible before and after treatment shows the complementarity of tissue conditioning and pre-prosthetic surgery, thus the added value of this technique in the management of a large EF. After the surgical resection of an EF, periodic controls are crucial to check not only the dentures adaptation and monitor bone resorption but also to detect an eventual recurrence of the EF.

## Conclusion

This case report shows the importance of the combination of tissue conditioning and pre-prosthetic surgery in the management of a large epulis fissuratum (EF). The originality of this procedure reside in the simulation of the surgical procedure on the secondary cast, the correction of the pre-existing prosthesis, and its use to guide postoperative tissue healing. This operating protocol, helped in optimizing the duration of the treatment, not to mention an increased patient comfort, by maintaining oral function during the treatment. The limitations of this technique is that it requires an experienced lab technician, one that can realize a precise surgical simulation on the secondary cast and correct the pre-existing dentures following the clinician´s exact directions. Patient collaboration is also a key factor, since the patient plays an active role in this protocol.
